# In silico analysis of the prognostic value of FAS mRNA in malignancies

**DOI:** 10.7150/jca.35614

**Published:** 2020-01-01

**Authors:** Zhigang Chen, Jun Wu, Hailin Xu, Xiuyan Yu, Ke Wang

**Affiliations:** 1 Department of Surgical Oncology, Second Affiliated Hospital, Zhejiang University School of Medicine, Hangzhou, China; 2 Key Laboratory of Tumor Microenvironment and Immune Therapy of Zhejiang Province, Hangzhou, China; 3 Department of General surgery, the First People's Hospital of Jiande, HangZhou, China

**Keywords:** FAS, biomarker, prognosis

## Abstract

**Background:** FAS is a classical death receptor involved in the FAS/FAS ligand (FASL) apoptosis pathway and plays a role in anti-tumor activity. Some studies have recently reported that FAS can serve as an oncogene that promotes tumor proliferation and maintains the stemness of tumor cells. Hence, its prognostic value in malignancies remains controversial.

**Methods:** we assessed the prognostic value of FAS mRNA in several types of tumors by online platforms including Kaplan-Meier Plotter and SurvExpress.

**Results:** FAS mRNA was associated with better overall survival (OS) in breast cancer (Hazard ratio (HR): 0.59 [0.47, 0.73]; p=1.5e-06), gastric cancer (HR: 0.65 [0.54, 0.77]; p=8e-07) and non-small-cell lung cancer (NSCLC) (HR: 0.78 [0.69, 0.89]; p=0.00016), especially in lung adenocarcinoma (HR: 0.64 [0.51, 0.81], p=1.7e-04), female lung cancer (HR:0.72 [0.57, 0.9], p=0.0049) and patients who have never smoked (HR: 0.39 [0.21, 0.7], p=0.0012). However, a high level of FAS mRNA expression indicated poorer OS in pancreatic cancer (HR: 1.33 [1.06, 1.66]; p=0.01) and acute myeloid leukemia (AML) (HR: 1.57 [1.02, 2.41], p=0.04). Additionally, FAS showed no prognostic value in renal carcinoma, head and neck carcinoma, hepatic cancer, ovarian cancer, colorectal cancer or glioblastoma. The results from the Cell Miner tool revealed that FAS expression was associated with the sensitivity of tumor cells to cabozantinib and erlotinib.

**Conclusions:** In summary, the dominant function of FAS may vary in different malignancies. FAS mRNA expression was correlated with better OS in breast cancer, gastric cancer and lung cancer, but worse OS in pancreatic cancer and AML. We also suggested that FAS mRNA expression could be a potential biomarker for cabozantinib and erlotinib.

## Introduction

FAS encodes the classical death receptor which belongs to the tumor necrosis factor receptor (TNFR) family. It is activated when bound with FASL (FAS ligand), and then the cytoplasm side tail of FAS recruits FAS-associated death domain (FADD), pro-caspase-8/10 and a negative regulator, the cellular FADD-like interleukin-1 beta converting enzyme (FLICE; caspase-8) inhibitory protein (c-FLIP). These elements together compose the death inducing signaling complex (DISC), which activates the downstream caspases and induces apoptosis 1-3. It has been reported that FAS plays a positive role in inhibiting tumor cell progression 4, 5. FAS has been identified as a biomarker for breast cancer with better prognosis 6, 7, acute myeloid leukemia (AML) 8, urothelial cancer 9 and lung cancer 10, 11. Furthermore, FAS agonist antibody significantly decreased the progression of the tumor in the mice model with transplanted with human B cell tumors 5. Targeting the FAS/FASL signal pathway via the FAS agonist antibody or FASL fusion is a promising therapeutic strategy 4.

Recently, FAS has been reported to be associated with tumor cell proliferation, invasion and migration. In addition, resistance of FAS associated apoptosis exists universally in many tumor cells. Then activated FAS pathway promotes the growth or metastasis of the tumor rather induces apoptosis 12, 13. The absence of FAS protein has been reported to be correlated with worse clinicopathological parameters in non-small-cell lung cancer (NSCLC)^14^ and hepatocellular carcinoma^15^.

Hence, the prognostic value of FAS in malignancies remains controversial because of the paradoxical role of FAS. Here we first investigated the association between FAS mRNA expression and prognosis in different tumors based on KM-Plotter and other online databases. Additionally, we tried to study the potential role of FAS in predicting the efficiency of target drugs including vemurafenib, dabrafenib, crizotinib, carbozantinib, erlotinib and afatinib.

## Material and Methods

### Study design

We estimated the association between FAS mRNA expression and prognosis in several malignancies using the following two databases: The Kaplan-Meier Plotter (http://www.kmplot.com//) 16 and SurvExpress (http://bioinformatica.mty.itesm.mx/SurvExpress) 17.

#### Kaplan-Meier Plotter

The recommended probe (204781_s_at) was chosen for evaluation. The survival curves and log-rank p values were obtained, and four types of malignancies including breast cancer, lung carcinoma, ovarian cancer and gastric cancer were investigated. Patients were divided into two groups by median expression level of FAS mRNA. The median expression levels of FAS in each type of tumor were also provided in Table S3.

#### SurvExpress

The probe of FAS with original (quantile-normalized) data was evaluated, and different probes for FAS were averaged per sample. The samples were divided by the median FAS mRNA expression. The hazard ratios with log-rank p values were calculated. The pooled results of datasets for each type of tumor were estimated by meta-analysis. The clinical data of tumors obtained from SurvExpress contained acute myeloid leukemia, pancreatic cancer, glioblastoma, colorectal cancer, head and neck carcinoma, hepatic cancer and renal carcinoma.

### Data analysis

The significant difference in survival was estimated using the Kaplan-Meier method between the FAS mRNA high expression group and the FAS mRNA low expression group. The hazard ratios (HRs) were estimated using the Cox regression analysis method, and the log-rank p value was calculated. A p<0.05 was considered statistically significant. The pooled HRs and p values of survival data from SurvExpress were estimated using RevMan version 5.3.

### FAS mRNA expression and drug sensitivity

The values of FAS mRNA expression (transcript log 2 intensities) in a panel of 60 diverse human cancer cell lines (NCI 60) and the activity values of several drugs targeting lung cancer, pancreatic cancer and gastric cancer were downloaded from the Cell Miner 60 website (https://discover.nci.nih.gov/ cellminer/). The original data of drug sensitivity was downloaded as the mean-centered log10 values of 50% growth inhibition. The value of FAS expression and drugs sensitivities were transformed to Z-scores (standard scores). The detailed process of this transformation was provided below; the value of the FAS expression and the drug sensitivities were obtained by subtracting the means of each and dividing them by the standard deviations. A linear regression analysis was used to estimate the association between FAS mRNA expression and the sensitivity of drugs. The coefficient of determination (R squared; R^2), regression coefficient (RC) and the p value were used to estimate the correlation and they were calculated using SPSS version 22.

## Results

### Prognosis of FAS expression in malignancies

**Figure 1:**
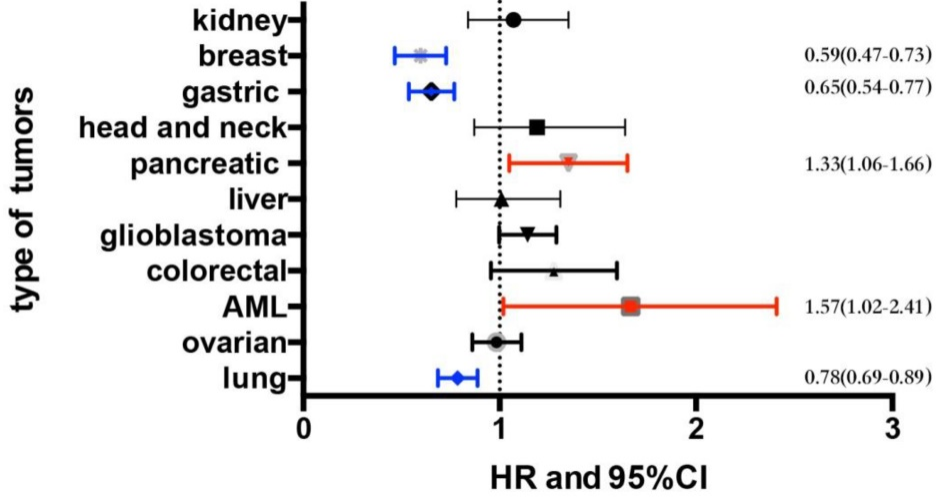
The summary of the association between FAS mRNA expression and OS in different types of malignancies.

We examined the prognostic values of the *FAS* mRNA expression in several malignancies including breast cancer, gastric cancer, non-small-cell lung cancer, pancreatic cancer, acute myeloid leukemia (AML), renal carcinoma, head and neck carcinoma, hepatic cancer, ovarian cancer, colorectal cancer and glioblastoma using the KM-Plotter and SurvExpress online databases. Multiple parameters are used to assess the prognosis of malignancies including overall survival (OS), relapse-free survival (RFS) and progression-free survival (PFS). OS means the time from the start of randomization to the death of any cause. RFS refers to the time from the start of randomization to the recurrence of the disease or the death of the patient due to disease progression. PFS indicates the time from the subject entering the trial to disease progression or died. The OS of different malignancies is shown in Figure 1 and Table S1. The median expression level in each type of tumor and a detailed distribution of the FAS mRNA expression are provided in Table S3. The detailed information of datasets that used for analyzing the prognostic value of FAS in each type of malignancy is provided in Table S4.

### Acute myeloid leukemia

**Figure 2:**
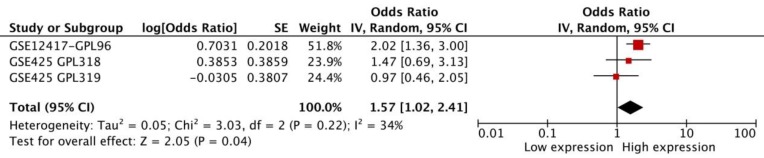
The meta-analysis of three datasets from SurvExpress about the HR and 95% confidence interval for OS of AML patients.

We performed a meta-analysis of 3 datasets from SurvExpress to assess the prognostic significance of FAS in AML. The results showed that FAS mRNA in AML was related with a worse OS (HR: 1.57 [1.02, 2.41], p=0.04, n=256 cases). There was no statistical heterogeneity in the meta-analysis (p=0.22) between different datasets (Figure 2).

### Breast cancer

**Figure 3:**
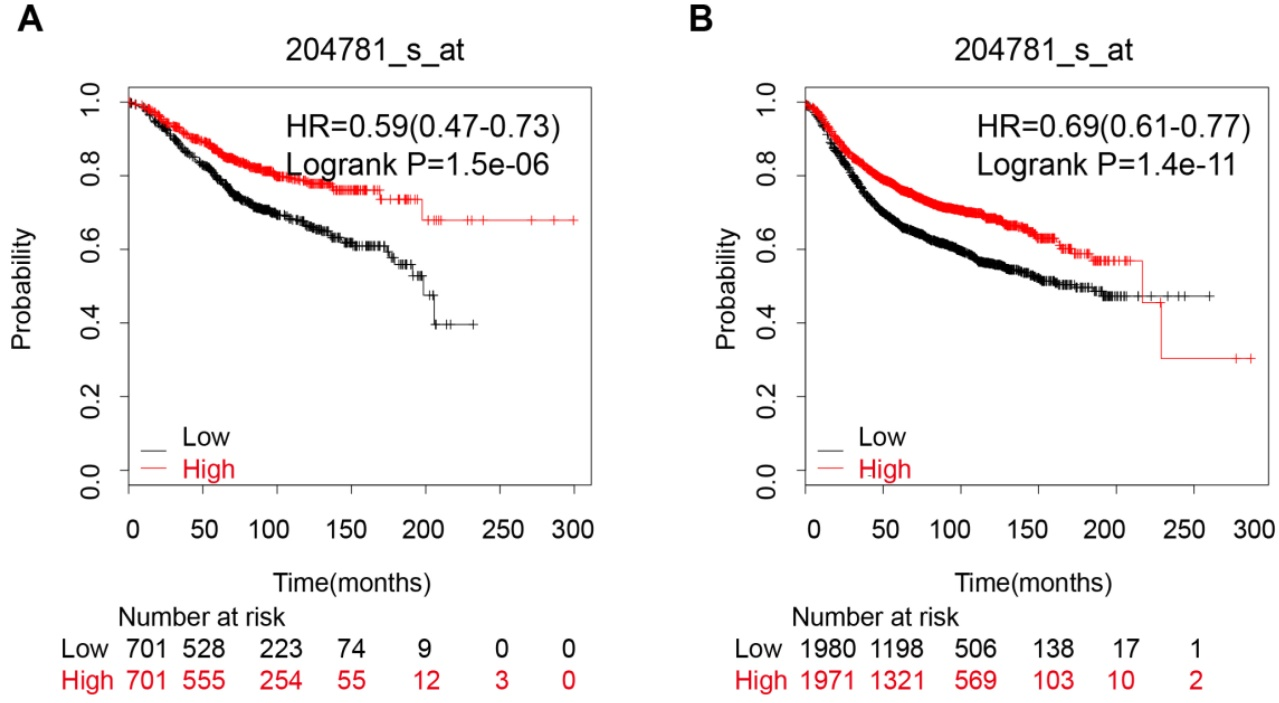
The correlation between FAS mRNA expression and prognosis of breast cancer patients. (A) The FAS mRNA expression is associated with a better OS in breast cancer patients; (B) The FAS mRNA expression is associated with a better RFS in breast cancer patients.

An analysis of 9 datasets pooled in KM-Plotter showed that high expression of FAS mRNA was connected with a better OS (HR:0.59 [0.47, 0.73], p=1.5e-06, n=1402 cases) and longer RFS (HR:0.69 [0.61, 0.77], p=1.4e-11, n=3951 cases) (Figure 3A and 3B).

### Lung carcinoma

**Figure 4:**
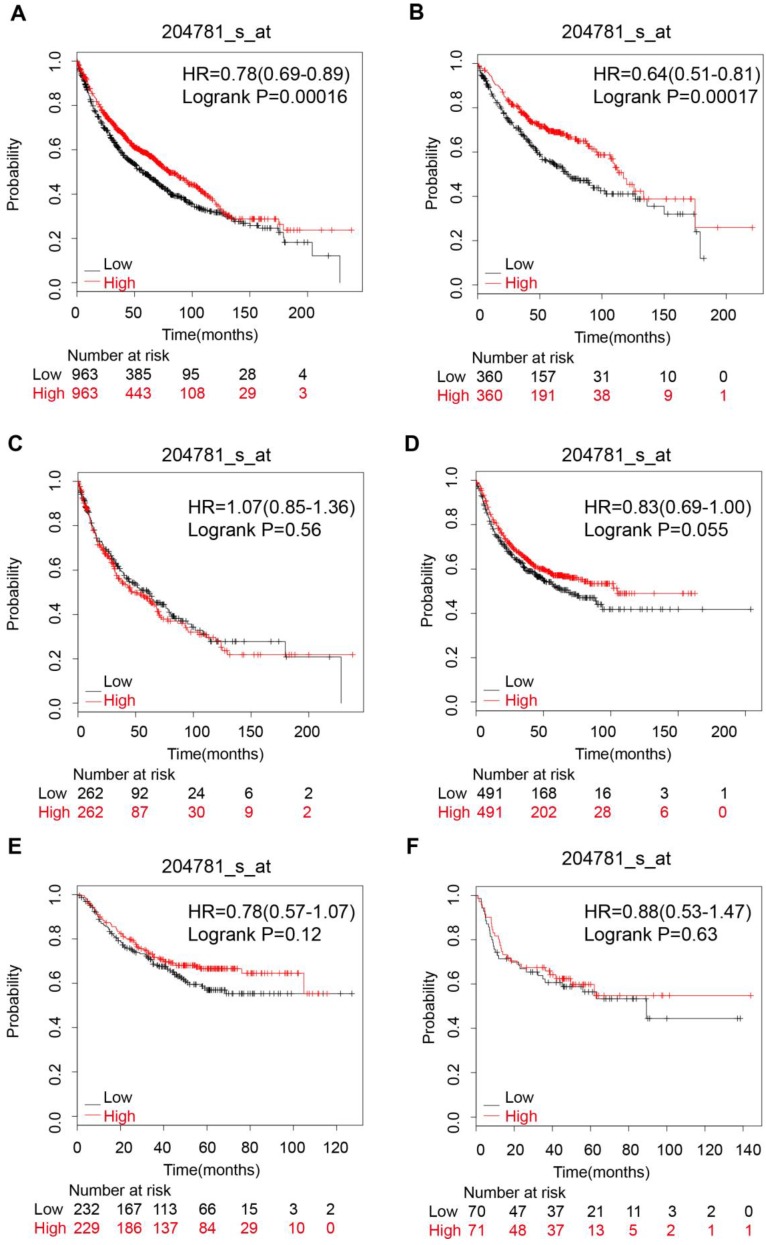
The prognostic value of FAS expression in lung cancer patients. (A) Survival curve for NSCLC patients; (B) Survival curve for lung adenocarcinoma patients; (C) Survival curve for lung squamous cells carcinoma patients; (D) Progression-free survival curve for NSCLC patients; (E) Progression-free survival curve for lung adenocarcinoma patients; (F) Progression-free survival curve for lung squamous cell carcinoma patients.

The pooled survival results of 12 datasets from KM-Plotter showed that FAS expression was significantly related with a better OS (HR: 0.78 [0.69, 0.89], p=1.6e-04, n=1926 cases) in NSCLC (Figure 4A). However, the significant correlation with OS only existed in lung adenocarcinoma (HR: 0.64 [0.51, 0.81], p=1.7e-04, n=720 cases) (Figure 4B), female lung cancer (HR: 0.72 [0.57 , 0.9], p=0.0049) (Table 1) and patients who had never smoked (HR: 0.39 [0.21, 0.7], p=0.0012) (Table 1) but not in lung squamous cell carcinoma (HR: 1.07 [0.85, 1.36], p=0.56) (Figure 4C), male lung cancer(HR: 0.93 [0.8, 1.09], p=0.4) and patients who had ever smoked (HR: 0.84 [0.68, 1.03], p=0.097) (Table 1). In addition, FAS expression was not associated with the PFS, regardless of histologic subtypes (Figure 4D,E,F Table 1).

### Gastric cancer

**Figure 5:**
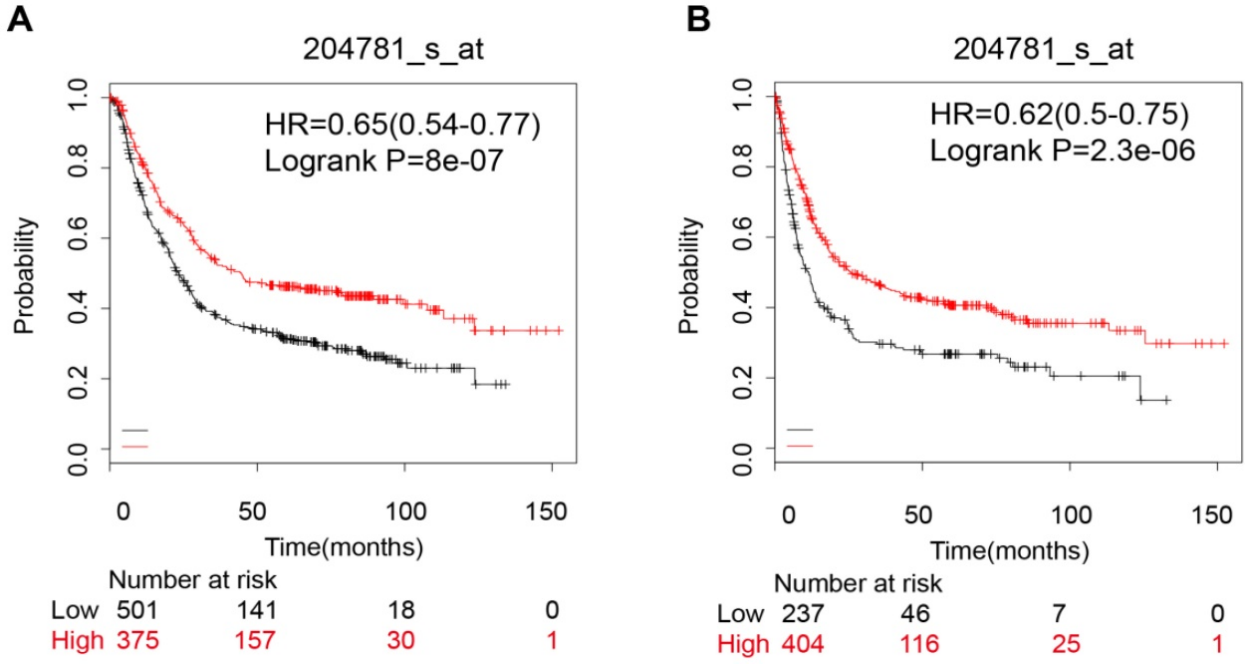
The prognostic value of FAS mRNA expression in gastric cancer patients. (A) The overall survival curve of all gastric cancer patients; (B) Progression-free survival curve for all gastric cancer patients.

Pooled results of 6 datasets from KM-Plotter indicated that the FAS mRNA high expression group had a better OS (OS, HR: 0.65 [0.54, 0.77], p=8.0e-07, n=876 cases). (Figure 5A). However, when we divided the samples into a HER-2 positive subgroup and a HER-2 negative subgroup, the significant difference was only evident in HER-2 negative group (OS, HR: 0.57 [0.45, 0.72], p=2.7e-06). The FAS mRNA expression was also related to the PFS in gastric cancer (HR: 0.62 [0.5, 0.75], p=2.30e-06) (Figure 5B). In addition, the FAS mRNA was not related with HER-2 status (HER-2 (-): HR: 0.53 [0.4, 0.69], p=1.90e-06; HER-2 (+): HR: 0.57 [0.4, 0.8], p=0.0013) (Table 2).

### Pancreatic cancer

**Figure 6:**
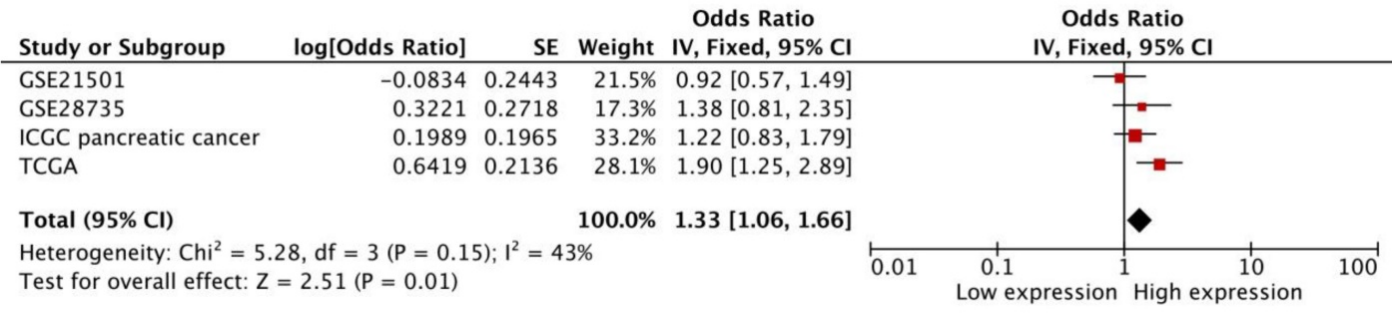
Meta-analysis of four datasets of pancreatic cancer patients. The expression of FAS mRNA is associated with worse OS in pancreatic cancer patients.

The pooled result was based on 4 datasets from SurvExpress, and heterogeneity did not exist (p=0.15). The high transcriptional expression of FAS was associated with a worse OS (HR:1.33 [1.06, 1.66], p=0.01, n=551 cases) (Figure 6).

### Other types of malignancies

The data from KM-Plotter showed that FAS mRNA expression was not correlated with OS and PFS in ovarian cancer (OS, HR: 0.98 [0.86, 1.11], p=0.74, n=1656 cases; PFS, HR: 0.98 [0.87, 1.12], p=0.8, n=1435 cases) (Table S2). In glioblastoma, the pooled result of 10 datasets from SurvExpress indicated that high expression of FAS mRNA showed a tendency to be associated with OS (HR: 1.13 [1, 1.29], p=0.06, n=1377 cases). The FAS expression was not related with the prognosis of renal carcinoma (OS, HR: 1.07 [0.84, 1.35], p=0.6, n=743 cases), hepatic cancer (OS, HR: 1.01 [0.78, 1.31], p=0.93, n=623 cases), head and neck carcinoma (OS, HR: 1.19 [0.87, 1.64], p=0.27, n=369 cases) and colorectal cancer (OS, HR: 1.25 [0.97, 1.61], p=0.08, n=741 cases) (Figure 1 and Table S1). The data of these four types of malignancies were based on the meta-analysis of datasets from SurvExpress, and all of them showed no significant heterogeneity (Figure S1 A-E).

### Correlation between drug sensitivity and FAS expression

**Figure 7:**
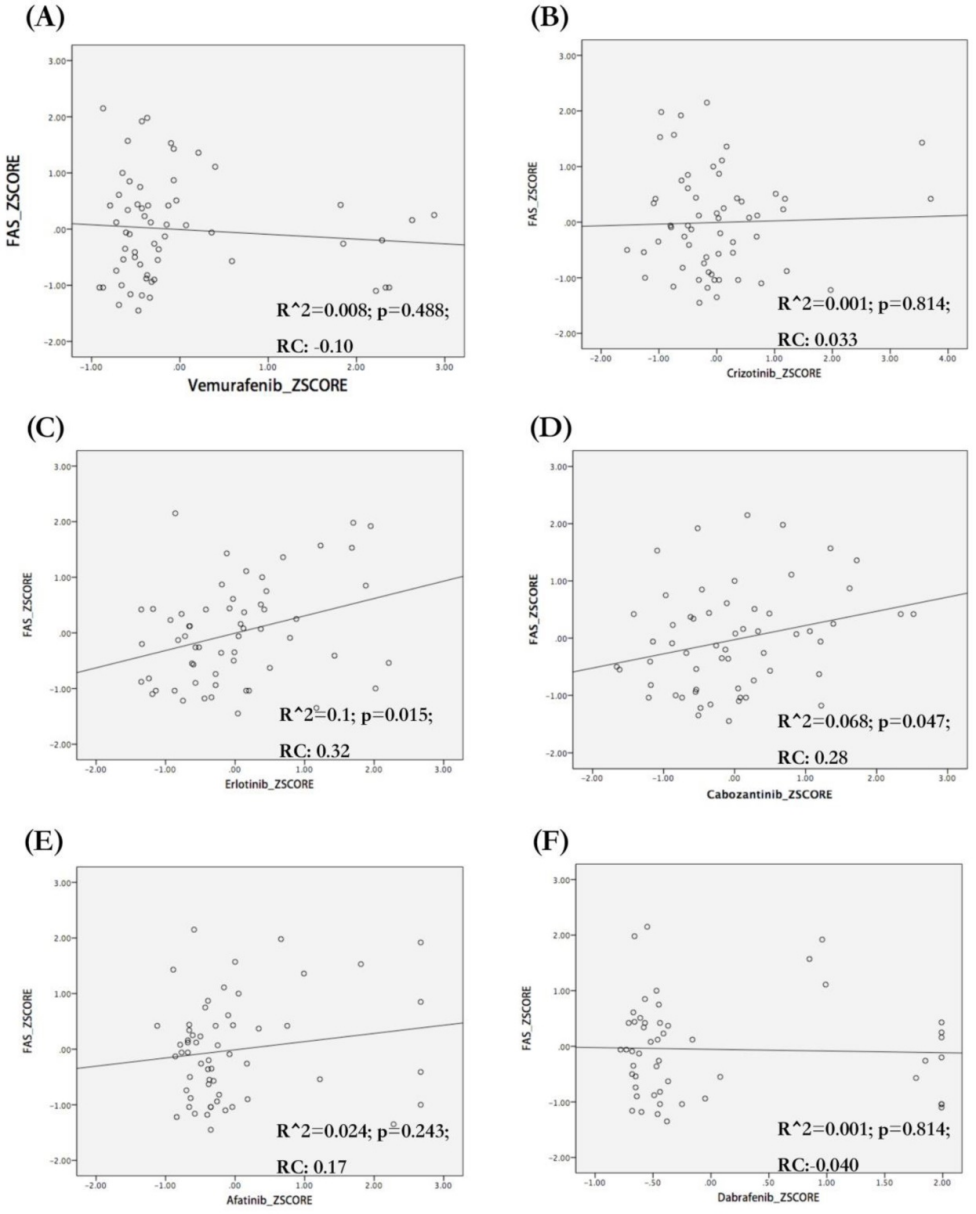
Association between FAS mRNA expression and sensitivity of different cell lines to target drugs. The regression figures of vemurafenib (A), crizotonib (B), erlotinib (C), cabozantinib (D), afatinib (E) and dabrafenib (F). The data were transformed to Z-Scores and were downloaded from Cell Miner Tools. Only the sensitivity of tumor cells to erlotinib (C. R square=0.1; p=0.015) and cabozantinib (D. R square=0.068; p=0.047) were correlated with FAS mRNA expression. The types of tumor cells with higher FAS mRNA expression seem to be more sensitive to erlotinib (C) and cabozantinib (D). RC: Regression coefficient; R^2: R squared (coefficient of determination).

We analyzed the correlation between FAS mRNA expression and the sensitivity of different tumor cell lines to target drugs for NSCLC (vemurafenib, dabrafenib, crizotinib and carbozantinib), pancreatic cancer (erlotinib) and breast cancer (afatinib). We observed that FAS mRNA expression was correlated with the sensitivity of tumor cells to erlotinib (R^2=0.1; p=0.015; RC=0.32) (Figure 7C) and carbozantinib (R^2=0.068; p=0.047; RC=0.28) (Figure 7D), but not related with vemurafenib, dabrafenib, crizotinib and afatinib (Figure 7A,B,E,F).

## Discussion

FAS, which belongs to TNFR/TNF family, was known as the trigger of the classical apoptosis pathway. It is an important factor that activates the downstream caspases and initiates the process of apoptosis 1-3, 18. It was reported that FAS protein was related to a better prognosis in breast cancer, acute myeloid leukemia, urothelial cancer and lung cancer. However, many studies have revealed that FAS-associated apoptosis resistance existed in most types of tumors 19, 20. In addition, FAS played roles in tumor growth, invasion and metastasis in addition to inducing apoptosis 12, 21. Therefore, the prognostic value of FAS in malignancies remains controversial. Although previous studies have analyzed the prognostic value of FAS, the sample size was small. In this study, we systematically investigated the association between FAS mRNA expression and prognosis in different tumors based on meta-analysis integrating a large number of microarray data. We found that FAS mRNA expression was correlated with better survival in breast cancer, gastric cancer and lung cancer, but it was associated with a worse prognosis in pancreatic cancer and AML; no relationship was found with kidney cancer, head and neck cancer, hepatic cancer, glioblastoma, colorectal cancer or ovarian cancer. However, our findings were based on the bioinformatics analyses of publicly available datasets. More data from clinical studies are warranted to verify these results.

Our study found that the cases with high expression of FAS had a significant better OS and RFS in breast cancer. Previous studies showed that the group that expressed FAS protein tended to have a longer DFS, less recurrence, better nodal status and smaller tumor size 22, 23. The lymph node negative cases were more likely to be FAS positive (70% versus 30%) 6. Our analysis of mRNA levels was also consistent with these results.

It has been reported that FAS on cell membranes decrease generally in NSCLC and mainly exist in the cytoplasm 11. FAS associated phosphatase 1 (FAP-1) can interact with the C-terminal of FAS and leave FAS in the cytoplasmic cytoskeleton 24.The loss of the cell membrane FAS was associated with advanced stages and lower survival rates 10, 11, 14. On the other hand, it was also found that stage III NSCLC patients who expressed FAS proteins had longer survival 25. The FAS in the cytoplasm was suggested to be a reason for apoptosis resistance 26. In this study, we found that the expression of FAS was associated with better OS in lung adenocarcinoma but not in lung squamous cell carcinoma. The FAS also only shows the prognostic value in females and the group with no smoking history. Coincidentally, it has been identified that female lung adenocarcinoma patients are more likely to be non-smokers^27^. It is still unknown why FAS only has a prognostic value in lung adenocarcinoma patients but not in lung squamous cell carcinoma patients.

Gastric cancer is another type of tumor that shows a connection between high expression of FAS mRNA and better survival in this study. However, previous studies have reported that the positive expression of FAS protein was correlated with disease progression, such as nodal invasion and advanced stages 28. Some studies reported that high expression of FAS meant better differentiation of tumor cells^29^ and FAS was down-regulated in gastric cancer^30^. Hence, the role of FAS in gastric cancer remains controversial. We included six datasets and 876 patients in this study and found that high expression of FAS mRNA indicated better survival.

It has been reported that the FAS-associated apoptosis resistance also existed in AML cells 8. Others have reported that the FAS positive expression group had a longer RFS after complete remission (CR), but it was not correlated with OS in M1 to M6 AML patients. However, no connection was showed between the FAS expression and CR rate 31. This study was based on unselected AML patients, and the follow-up did not begin after CR. We suggest that high expression of FAS mRNA in unselected AML patients is associated with worse OS.

Previous studies have indicated that the FAS transcriptional expression was higher in metastases than it in the primary lesion of pancreatic ductal adenocarcinoma. The high FAS expression was accompanied by up-regulation of epithelial-mesenchymal transition genes, which were responsible for maintaining stemness and initiating metastasis 32. Furthermore, FAS can induce pro-inflammatory responses, which resulted in tumor cell proliferation, invasion and metastasis^33^. Therefore, FAS expression is significantly correlated with worse prognosis. In this study, we also found that the high expression of FAS mRNA was associated with a worse prognosis.

Surprisingly, we found that the tumor cells with higher FAS mRNA expression tend to be more sensitive to cabozantinib and erlotinib. Cabozantinib is an oral tyrosine kinase receptor inhibitor, which is recommended for advanced NSCLC patients with RET rearrangement. It was reported that the FAS/c-Met interaction can reduce the formation of DISC. The inhibition of c-Met may up-regulate the DISC and activate the FAS-associated apoptosis pathway expression^34^. The AXL can also interact with the TNFR and block the recruitment of caspase-8 to the DISC. These findings can partially explain the predictive value of FAS for cabozantinib^35^. Erlotinib is an epidermal growth factor receptor (EGFR) tyrosine kinase inhibitor (TKI) and has been widely used in EGFR mutant advanced NSCLC patients 36 and advanced pancreatic cancer patients 37. In fact, the EGFR inhibitor can up-regulate the FAS-associated apoptosis without affecting the expression of FAS. The inhibition of EGFR could down-regulate the c-FLIP expression 38. Overall, FAS seems to be a potential biomarker for anticipating the efficacies of cabozantinib and erlotinib. Deeper studies are warranted to rigorously investigate the underlying mechanisms.

In summary, FAS has a dual and complicated role in tumors, including both anti-cancer and cancer promotion functions. FAS mRNA expression can be used as a prognostic marker in certain cancers including breast cancer, gastric cancer, lung cancer, pancreatic cancer and AML. It also has the potential to be a sensitive marker for cabozantinib and erlotinib.

## Supplementary Material

Supplementary figures and tables.

## Figures and Tables

**Table 1:** The prognostic value of FAS mRNA expression in different subgroups of lung cancer patients

Subgroups	OS		PFS	
	HR and 95%CI	P value	HR and 95%CI	P value
**all**	0.78 (0.69 - 0.89)	0.00016	0.83 (0.69 - 1)	0.055
**Gender**				
female	0.72 (0.57 - 0.9)	0.0049	0.92 (0.69 - 1.23)	0.58
male	0.93 (0.8 - 1.09)	0.4	0.85 (0.66 - 1.1)	0.23
**Smoking history**				
never	0.39 (0.21 - 0.7)	0.0012	0.56 (0.35 - 0.92)	0.019
had ever	0.84 (0.68 - 1.03)	0.097	0.86 (0.67 - 1.1)	0.22
**Stage**				
1	0.52 (0.39 - 0.68)	1.90E-06	0.66 (0.43 - 1.03)	0.067
2	0.75 (0.52 - 1.08)	0.12	1.64 (0.97 - 2.77)	0.061
3	0.99 (0.57 - 1.7)	0.96	/	/
4	/	/	/	/
**Grade**				
I	1.1 (0.77 - 1.58)	0.6	0.92 (0.6 - 1.43)	0.72
II	0.83 (0.61 - 1.14)	0.25	0.76 (0.5 - 1.15)	0.19
III	1.12 (0.58 - 2.18)	0.73	1.44 (0.64 - 3.23)	0.37
**Histology**				
adenocarcinoma	0.64 (0.51 - 0.81)	0.00017	0.78 (0.57 - 1.07)	0.12
squamous cell carcinoma	1.07 (0.85 - 1.36)	0.56	0.88 (0.53 - 1.47)	0.63
**Chemotherapy**				
yes	0.82 (0.55 - 1.23)	0.34	0.78 (0.51 - 1.18)	0.23
no	0.76 (0.54 - 1.06)	0.11	0.78 (0.53 - 1.15)	0.2
**Radiotherapy**				
yes	1.05 (0.62 - 1.79)	0.86	0.75 (0.44 - 1.27)	0.28
no	0.85 (0.6 - 1.22)	0.39	0.79 (0.54 - 1.15)	0.22

**Table 2:** The prognostic value of FAS mRNA expression in different subgroups of gastric cancer patients

Subgroups	OS		PFS	
	HR and 95%CI	P value	HR and 95%CI	P value
**all**	0.65(0.54-0.77)	8.00E-07	0.62 (0.5 - 0.75)	2.30E-06
**Gender**				
female	0.55 (0.38 - 0.79)	0.0012	0.58 (0.39 - 0.86)	6.30E-03
male	0.66 (0.53 - 0.82)	0.00015	0.58 (0.44 - 0.75)	3.40E-05
**Stage**				
1	0.28 (0.1 - 0.79)	0.01	0.23 (0.07 - 0.72)	0.0063
2	0.48 (0.27 - 0.88)	0.015	0.49 (0.27 - 0.9)	0.018
3	0.58 (0.41 - 0.8)	0.0011	0.64 (0.42 - 0.97)	0.034
4	0.65 (0.42 - 1.01)	0.056	0.86 (0.58 - 1.26)	0.34
**Differentiation**				
poorly	0.61 (0.39 - 0.97)	0.033	0.67 (0.41 - 1.1)	0.11
moderately	0.63 (0.31 - 1.25)	0.18	0.58 (0.31 - 1.08)	0.083
well	/	/	/	/
**HER-2 status**				
positive	0.77 (0.59 - 1.01)	0.06	0.57 (0.4 - 0.8)	0.0013
negative	0.57 (0.45 - 0.72)	2.7e-06	0.53 (0.4 - 0.69)	1.90E-06

## References

[1] Scott FL, Stec B, Pop C, Dobaczewska MK, Lee JJ, Monosov E (2009). The Fas-FADD death domain complex structure unravels signalling by receptor clustering. Nature.

[2] Peter ME, Hadji A, Murmann AE, Brockway S, Putzbach W, Pattanayak A (2015). The role of CD95 and CD95 ligand in cancer. Cell Death Differ.

[3] Fu Q, Fu TM, Cruz AC, Sengupta P, Thomas SK, Wang S (2016). Structural Basis and Functional Role of Intramembrane Trimerization of the Fas/CD95 Death Receptor. Mol Cell.

[4] Villa-Morales M, Fernandez-Piqueras J (2012). Targeting the Fas/FasL signaling pathway in cancer therapy. Expert Opin Ther Targets.

[5] Trauth BC, Klas C, Peters AM, Matzku S, Moller P, Falk W (1989). Monoclonal antibody-mediated tumor regression by induction of apoptosis. Science (New York, NY).

[6] Mottolese M, Buglioni S, Bracalenti C, Cardarelli MA, Ciabocco L, Giannarelli D (2000). Prognostic relevance of altered Fas (CD95)-system in human breast cancer. Int J Cancer.

[7] Reimer T, Herrnring C, Koczan D, Richter D, Gerber B, Kabelitz D (2000). FasL:Fas ratio-a prognostic factor in breast carcinomas. Cancer research.

[8] Tourneur L, Delluc S, Levy V, Valensi F, Radford-Weiss I, Legrand O (2004). Absence or low expression of fas-associated protein with death domain in acute myeloid leukemia cells predicts resistance to chemotherapy and poor outcome. Cancer research.

[9] Yamana K, Bilim V, Hara N, Kasahara T, Itoi T, Maruyama R (2005). Prognostic impact of FAS/CD95/APO-1 in urothelial cancers: decreased expression of Fas is associated with disease progression. British journal of cancer.

[10] Li Y, Xu KP, Jiang D, Zhao J, Ge JF, Zheng SY (2015). Relationship of Fas, FasL, p53 and bcl-2 expression in human non-small cell lung carcinomas. International journal of clinical and experimental pathology.

[11] Myong NH (2005). Tissue microarray analysis of Fas and FasL expressions in human non-small cell lung carcinomas; with reference to the p53 and bcl-2 overexpressions. J Korean Med Sci.

[12] Zheng HX, Cai YD, Wang YD, Cui XB, Xie TT, Li WJ (2013). Fas signaling promotes motility and metastasis through epithelial-mesenchymal transition in gastrointestinal cancer. Oncogene.

[13] Chen L, Park SM, Tumanov AV, Hau A, Sawada K, Feig C (2010). CD95 promotes tumour growth. Nature.

[14] Koomagi R, Volm M (1999). Expression of Fas (CD95/APO-1) and Fas ligand in lung cancer, its prognostic and predictive relevance. Int J Cancer.

[15] Ito Y, Monden M, Takeda T, Eguchi H, Umeshita K, Nagano H (2000). The status of Fas and Fas ligand expression can predict recurrence of hepatocellular carcinoma. British journal of cancer.

[16] Gyorffy B, Lanczky A, Eklund AC, Denkert C, Budczies J, Li Q (2010). An online survival analysis tool to rapidly assess the effect of 22,277 genes on breast cancer prognosis using microarray data of 1,809 patients. Breast Cancer Res Treat.

[17] Aguirre-Gamboa R, Gomez-Rueda H, Martinez-Ledesma E, Martinez-Torteya A, Chacolla-Huaringa R, Rodriguez-Barrientos A (2013). SurvExpress: an online biomarker validation tool and database for cancer gene expression data using survival analysis. PLoS ONE.

[18] Ashkenazi A (2002). Targeting death and decoy receptors of the tumour-necrosis factor superfamily. Nature reviews Cancer.

[19] Yamada N, Noguchi S, Kumazaki M, Shinohara H, Miki K, Naoe T (2014). Epigenetic regulation of microRNA-128a expression contributes to the apoptosis-resistance of human T-cell leukaemia jurkat cells by modulating expression of fas-associated protein with death domain (FADD). Biochimica et biophysica acta.

[20] Liu K (2010). Role of apoptosis resistance in immune evasion and metastasis of colorectal cancer. World J Gastrointest Oncol.

[21] Peter ME, Budd RC, Desbarats J, Hedrick SM, Hueber AO, Newell MK (2007). The CD95 receptor: apoptosis revisited. Cell.

[22] Sjostrom J, Blomqvist C, von Boguslawski K, Bengtsson NO, Mjaaland I, Malmstrom P (2002). The predictive value of bcl-2, bax, bcl-xL, bag-1, fas, and fasL for chemotherapy response in advanced breast cancer. Clinical cancer research: an official journal of the American Association for Cancer Research.

[23] Bebenek M, Dus D, Kozlak J (2006). Fas and Fas ligand as prognostic factors in human breast carcinoma. Medical science monitor: international medical journal of experimental and clinical research.

[24] Ivanov VN, Bergami PL, Maulit G, Sato TA, Sassoon D, Ronai Ze (2003). FAP-1 Association with Fas (Apo-1) Inhibits Fas Expression on the Cell Surface. Molecular and cellular biology.

[25] Uramoto H, Osaki T, Inoue M, Taga S, Takenoyama M, Hanagiri T (1999). Fas expression in non-small cell lung cancer: its prognostic effect in completely resected stage III patients. Eur J Cancer.

[26] Mojtahedzadeh S, Hashimoto S, Nakashima Y, Koga T, Matsuo Y, Yoshino I (2002). Clinicopathologic relevance of apoptotic and proliferative factors in human lung adenocarcinoma: Fas expression correlates with the histologic subtype, but not with the degree of apoptosis. Pathology, research and practice.

[27] Akazawa Y, Satoh H, Takeda YY, Takiguchi K, Ishikawa H, Ohtsuka M (2003). Significantly lower rate of smoking in female compared to male patients with lung adenocarcinoma. Eur J Cancer Care (Engl).

[28] Gryko M, Guzinska-Ustymowicz K, Kisluk J, Cepowicz D, Kemona A, Kedra B (2014). High Fas expression in gastric carcinoma cells as a factor correlating with the occurrence of metastases to regional lymph nodes. Adv Med Sci.

[29] Li Q, Peng J, Li XH, Liu T, Liang QC, Zhang GY (2010). Clinical significance of Fas and FasL protein expression in gastric carcinoma and local lymph node tissues. World J Gastroenterol.

[30] Wang X, Fu Z, Chen Y, Liu L (2017). Fas expression is downregulated in gastric cancer. Mol Med Rep.

[31] Min YJ, Lee JH, Choi SJ, Chi HS, Lee JS, Kim WK (2004). Prognostic significance of Fas (CD95) and TRAIL receptors (DR4/DR5) expression in acute myelogenous leukemia. Leuk Res.

[32] Teodorczyk M, Kleber S, Wollny D, Sefrin JP, Aykut B, Mateos A (2015). CD95 promotes metastatic spread via Sck in pancreatic ductal adenocarcinoma. Cell Death Differ.

[33] Roder C, Trauzold A, Kalthoff H (2011). Impact of death receptor signaling on the malignancy of pancreatic ductal adenocarcinoma. Eur J Cell Biol.

[34] Smyth LA, Brady HJ (2005). cMet and Fas receptor interaction inhibits death-inducing signaling complex formation in endothelial cells. Hypertension.

[35] Hong J, Belkhiri A (2013). AXL mediates TRAIL resistance in esophageal adenocarcinoma. Neoplasia (New York, NY).

[36] Paik PK, Varghese AM, Sima CS, Moreira AL, Ladanyi M, Kris MG (2012). Response to erlotinib in patients with EGFR mutant advanced non-small cell lung cancers with a squamous or squamous-like component. Mol Cancer Ther.

[37] Moore MJ, Goldstein D, Hamm J, Figer A, Hecht JR, Gallinger S (2007). Erlotinib plus gemcitabine compared with gemcitabine alone in patients with advanced pancreatic cancer: a phase III trial of the National Cancer Institute of Canada Clinical Trials Group. Journal of clinical oncology: official journal of the American Society of Clinical Oncology.

[38] Iwase M, Takaoka S, Uchida M, Yoshiba S, Kondo G, Watanabe H (2008). Epidermal growth factor receptor inhibitors enhance susceptibility to Fas-mediated apoptosis in oral squamous cell carcinoma cells. Oral Oncol.

