# Supplementary material for: In silico analysis of the prognostic value of FAS mRNA in malignancies
Source: J Cancer. 2020 Jan 1;11(3):542–50. doi: 10.7150/jca.35614 (PMC6959050; doi:10.7150/jca.35614)
Supplement: Supplementary file 1 — Supplementary figures and tables. [file jcav11p0542s1.pdf]

Supplementary files

Table S1. Associations between FAS mRNA expression and overall survival in different types of malignancies. HR: hazard ratio; CI: confidence interval.

Type of malignancies	Overall survival	
	HR and 95% CI	p value
kidney cancer	1.07(0.84-1.35)	0.6
breast cancer	0.59 (0.47 − 0.73)	1.5e−06
gastric cancer	0.65(0.54-0.77)	8.00E-07
head and neck cancer	1.19(0.87-1.64)	0.27
pancreatic cancer	1.33(1.06-1.66)	0.01
liver cancer	1.01(0.78-1.31)	0.93
glioblastoma	1.14(1-1.29)	0.06
colorectal carcinoma	1.25(0.97-1.61)	0.08
acute myeloid leukemia	1.57(1.02-2.41)	0.04
ovarian cancer	0.98 (0.86 − 1.11)	0.74
lung cancer	0.78 (0.69 − 0.89)	0.00016

Table S2. The prognostic value of FAS mRNA expression in subgroups of ovarian cancer patients. HR: hazard ratio; CI: confidence interval.

Subgroups	Overall survival		Progression-free survival	
	HR and 95%CI	p value	HR and 95%CI	p value
all	0.98 (0.86 − 1.11)	0.74	0.98 (0.87 − 1.12)	0.8
**Stage**				
1	0.35 (0.1 − 1.31)	0.1	0.68 (0.23 − 2.05)	0.49
2	1.85 (0.6 − 5.68)	0.28	1.27 (0.65 − 2.5)	0.48
1+2	0.76 (0.35 − 1.67)	0.5	0.74 (0.42 − 1.32)	0.3
3	0.96 (0.82 − 1.13)	0.62	1.04 (0.89 − 1.21)	0.62
4	0.79 (0.55 − 1.14)	0.21	0.86 (0.59 − 1.24)	0.42
3+4	0.95 (0.82 − 1.1)	0.49	0.99 (0.86 − 1.14)	0.87
**Grade**				
I	0.84 (0.33 − 2.15)	0.72	/	/
I+II	0.93 (0.7 − 1.24)	0.63	1.16 (0.88 − 1.54)	0.3
III	0.97 (0.83 − 1.15)	0.75	1.01 (0.85 − 1.19)	0.93
IV	/	/	/	/
**p53 status**				
p53mut	1.12 (0.9 − 1.41)	0.31	1.07 (0.86 − 1.34)	0.54
p53wild	1.18 (0.67 − 2.08)	0.57	0.95 (0.56 − 1.6)	0.85
**Histology**				
endometrioid	/	/	0.7 (0.28 − 1.79)	0.46
serous	0.97 (0.83 − 1.12)	0.65	1 (0.87 − 1.16)	0.98

Table S3. The summary of median expression level of FAS for each type of malignancy.

<table>
<tr><td rowspan="2">Type of malignancies</td><td colspan="2">Median expression value (Expression range of the probe)</td></tr>
<tr><td>Overall survival</td><td>Relapse-free survival</td></tr>
<tr><td>breast cancer</td><td>413(7-2457)</td><td>393(7-2541)</td></tr>
<tr><td rowspan="2">Type of malignancies</td><td colspan="2">Median expression value (Expression range of the probe)</td></tr>
<tr><td>Overall survival</td><td>Progression-free survival</td></tr>
<tr><td>lung cancer</td><td>436(23-2531)</td><td>498(22-2531)</td></tr>
<tr><td>ovarian cancer</td><td>431(5-2298)</td><td>427(4-4749)</td></tr>
<tr><td>gastric cancer</td><td>531(57-4131)</td><td>367(57-3917)</td></tr>
<tr><td rowspan="2">Type of malignancies</td><td colspan="2">Median expression value (Expression range of the probe)</td></tr>
<tr><td colspan="2">Overall survival</td></tr>
<tr><td>renal carcinoma</td><td colspan="2">16(0.06-72)</td></tr>
<tr><td>head and neck carcinoma</td><td colspan="2">666(100-4186)</td></tr>
<tr><td>pancreatic cancer</td><td colspan="2">3(0.3-92)</td></tr>
<tr><td>hepatic cancer</td><td colspan="2">617(16-12855)</td></tr>
<tr><td>colorectal cancer</td><td colspan="2">5(0.3-2048)</td></tr>
<tr><td>glioblastoma</td><td colspan="2">98(0.47-20171)</td></tr>
<tr><td>acute myeloid leukemia</td><td colspan="2">369(0.4-1305)</td></tr>
</table>

Table S4. The detailed information of datasets used for analyzing the prognostic value of FAS.

breast cancer	Overall survival (OS)	Relapse-free survival (RFS)	acute myeloid leukemia	OS
	GSE1456	E-MTAB-365		GSE12417-GPL96
	GSE16446	GSE11121		GSE425-GPL318
	GSE16716	GSE12093		GSE425-GPL319
	GSE20271	GSE12276	head and neck cancer	OS
	GSE20685	GSE1456		GSE26549
	GSE20711	GSE16391		TCGA
	GSE3494	GSE16446	pancreatic cancer	OS
	GSE37946	GSE16716		TCGA
	GSE42568	GSE17705		GSE21501
	GSE45255	GSE17907		GSE28735
	GSE7390	GSE19615		ICGC
		GSE20271	hepatic cancer	OS
		GSE2034		GSE10143
		GSE20685		GSE10186
		GSE20711		TCGA
		GSE21653	colorectal cancer	OS
		GSE2603		GSE12945
		GSE26971		GSE24550
		GSE2990		GSE28722
		GSE31519		GSE30378
		GSE3494		GSE41258
		GSE37946		TCGA
		GSE42568	glioblastoma	OS
		GSE45255		BROAD
		GSE4611		GSE13041 GPL96
		GSE4922		GSE13041 GPL570
		GSE5327		GSE16011
		GSE6532		GSE2817
		GSE7390		GSE42669
		GSE9195		GSE4381
ovarian	OS	Progression-free survival (PFS)		GSE4412 GPL96
	GSE14764	GSE14764		GSE7696
	GSE15622	GSE15622		TCGA

	GSE18520	GSE26193	renal cancer	OS
	GSE19829	GSE26712		GSE29609
	GSE23554	GSE30161		GSE3538
	GSE26193	GSE32062		Poustka Sultmann Kidney
	GSE26712	GSE51373		TCGA
	GSE27651	GSE63885		
	GSE30161	GSE65986		
	GSE3149	GSE9891		
	GSE32062	TCGA		
	GSE63885			
	GSE9891			
	TCGA			
lung cancer	OS	PFS		
	CaArray	CaArray		
	GSE14814	GSE29013		
	GSE19188	GSE31210		
	GSE29013	GSE31908		
	GSE30219	GSE50081		
	GSE31210	GSE8894		
	GSE3141			
	GSE31908			
	GSE37745			
	GSE4573			
	GSE50081			
	TCGA			
gastric cancer	OS	PFS		
	GSE14210	GSE14210		
	GSE15459	GSE15459		
	GSE22377	GSE22377		
	GSE29272	GSE62254		
	GSE38749			
	GSE51105			
	GSE62254			

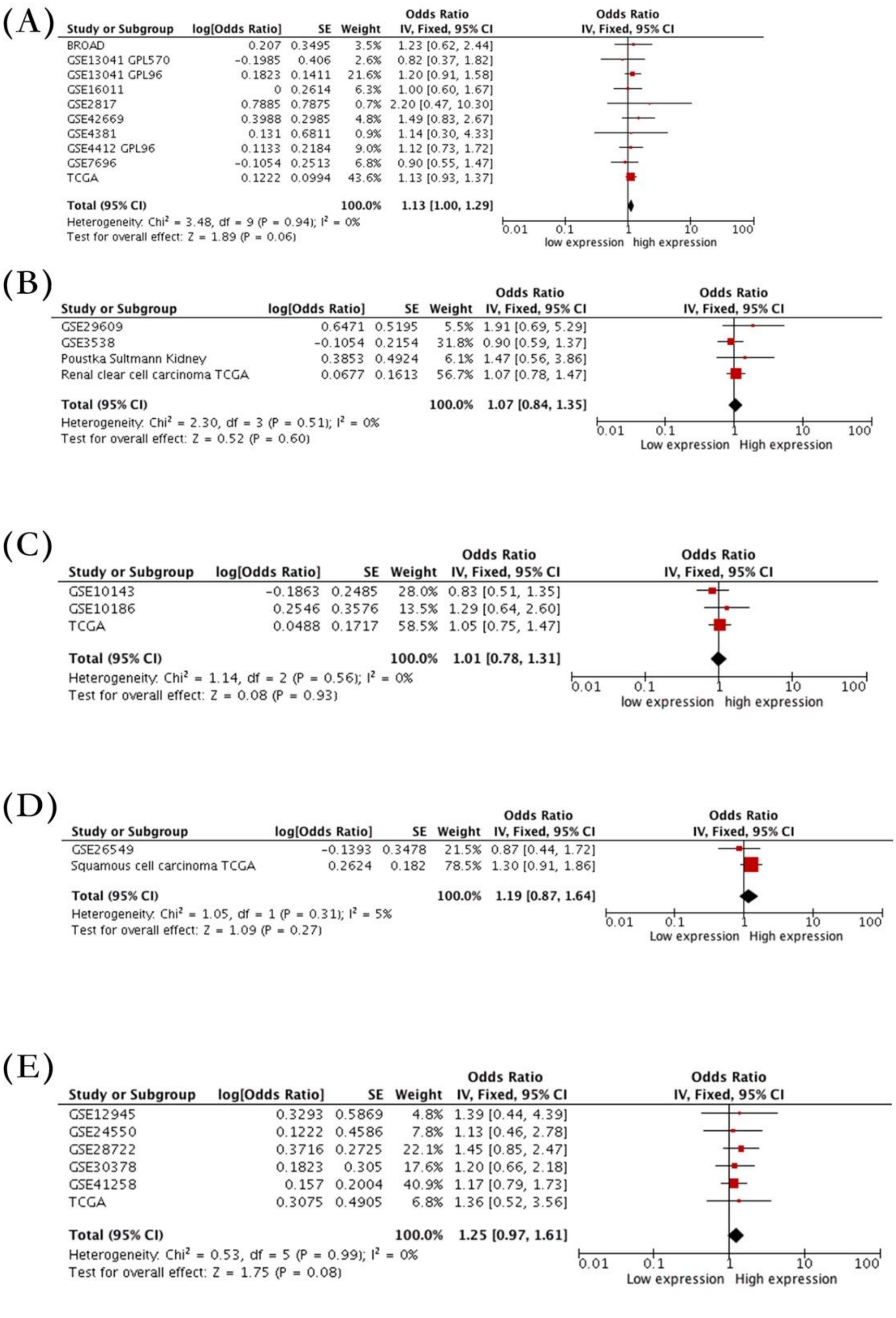

Figure S1. The pooled overall survival results from SurvExpress of five types of malignancies. The meta-analysis results of glioblastoma (A), renal cancer (B), hepatic cancer (C), head and neck carcinoma (D) and colorectal cancer (E).
